# Lead exposure is associated with increased lead bioaccumulation and a decline in semen quality: a systematic review and meta-analysis

**DOI:** 10.5935/1518-0557.20250163

**Published:** 2025

**Authors:** Victoria Ifeoluwa Adisa, Precious Jesutofunmi Ashonibare, Cecilia Adedeji Adegbola, Tunmise Maryanne Akhigbe, Oluwakemi Rebecca Kolawole, Isaac Ayomide Omole, Fabrael Batale Fidelis, Adetomiwa Ezekiel Adeogun, Rebecca Promise Oluwole, Bolaji Aderibigbe Akorede, Babatunde David Ogunkola, Adebayo Oluwafemi Adekunle, Suliat Adenike Hassan, Sulaiman Shuaibu Mansur, Sulaimon Bayonle Ajeigbe, Precious Adeoye Oyedokun, Victory Jesutoyosi Ashonibare, Olayinka Emmanuel Adelowo, Racheal Ibukun Oyesetan, Ayoola Abimbola Oladipo, Roland Eghoghosoa Akhigbe

**Affiliations:** 1 Department of Physiology, Ladoke Akintola University of Technology, Ogbomoso, Oyo State, Nigeria; 2 Reproductive Biology and Toxicology Research Laboratory, Oasis of Grace Hospital, Osogbo, Osun State, Nigeria; 3 Department of Agronomy, Osun State University, Ejigbo, Osun State, Nigeria; 4 Department of Biochemistry, Ahmadu Bello University, Zaria, Kaduna State, Nigeria; 5 Department of Physiology, Babcock University, Ilishan Remo, Ogun State, Nigeria; 6 Regions Hospital, Enugu, Enugu State, Nigeria; 7 Department of Curriculum and Instruction (Science Education), University of Wyoming, USA; 8 Department of Biological Sciences, Northern Arizona University, Flagstaff, AZ, USA; 9 Department of Biochemistry, Dokuz Eylul University, Izmir, Turkey; 10 Department of Community Health, Obafemi Awolowo University, Ile-Ife, Osun State, Nigeria; 11 Cardiovascular Regenerative Medicine & Tissue Engineering 3D Lab, Department of Cardiovascular Surgery and Research Group for Experimental Surgery, Heinrich Heine University, Medical Faculty, Düsseldorf, Germany

**Keywords:** endocrine disruptors, heavy metals, lead, male infertility, semen, testosterone

## Abstract

**Objective:**

This systematic review and meta-analysis aimed to assess the impact and associated mechanisms of lead on human semen quality.

**Methods:**

A systematic search was conducted from March 18^th^ to April 30^th^, 2024, utilizing Google Scholar, PubMed, and Scopus, and applying the PECOS model to identify relevant studies.

**Results:**

A total of seventeen studies fulfilled the inclusion criteria. The results of our analysis indicated that blood lead levels were markedly elevated in men exposed to lead compared to control subjects (SMD -7.06 [95% CI: -9.03, -5.08], p<0.00001), with analogous results observed for semen lead levels (SMD -3.42 [95% CI: -5.22, -1.62], p=0.0002). Lead exposure was linked to significant decreases in ejaculate volume (SMD 0.81 [95% CI: 0.16, 1.45], p=0.02), sperm count (SMD 2.10 [95% CI: 1.11, 3.09], p<0.0001), sperm concentration (SMD 0.77 [95% CI: 0.09, 1.44], p=0.03), and total motility (SMD 2.20 [95% CI: 1.28, 3.11], p<0.00001), as well as an increase in abnormal sperm morphology (SMD -3.29 [95% CI: -4.87, -1.71], p<0.0001). While reductions in testosterone levels and elevations in semen malondialdehyde were noted, these changes did not reach statistical significance.

**Conclusions:**

This study demonstrates that lead exposure is associated with reduced sperm quality. The present findings highlight the urgent need for strategies to reduce lead exposure and emphasize the importance of further research into potential mitigating interventions.

## INTRODUCTION

Lead, a widespread environmental contaminant, poses significant risks to human health. Although its adverse effects, such as adverse effects on the nervous system and heart, are widely recognized, its impact on male reproductive health has received increasing attention. Sources of lead exposure include environmental and occupational, smoking, and lead-containing cosmetics, jewelry, and diets such as root vegetables (Lasisi-Sholola *et al.*, 2024). Due to its widespread use in industrial activities and consumer products (Besong *et al.*, 2023a; 2023b; Farag *et al.*, 2024), exposure to lead is unavoidable. Its ubiquitous presence in the environment raises significant public health concerns regarding the potential for reduced sperm quality in affected communities. According to the World Health Organization (WHO), lead exposure contributes significantly to the global burden of disease, including 2.5% of ischemic heart disease cases, 2.4% of stroke cases, and 12.4% of unexplained cognitive developmental disorders cases (WHO - World Health Organization, 2024).

Lead-induced toxicity stems from its capacity to accumulate within male reproductive organs, such as the testes and epididymis, where it interferes with normal physiological functions (Besong *et al.*, 2024). Lead induces oxidative stress by generating reactive oxygen species (ROS) (Obiwulu, 2019) and undermines antioxidant defenses, resulting in cellular and molecular damage (Besong *et al.*, 2023a; 2023b; 2024). These processes can adversely affect sperm production and quality, as demonstrated by decreased sperm count, motility, and normal morphology (Balali-Mood *et al.*, 2021) and lower testosterone levels (Bentaiba *et al.*, 2023); thus contributing to a decrease in male fertility. However, the precise mechanisms through which lead exposure influences semen quality, including the roles of bioaccumulation, hormone disruption, and oxidative stress, remain inadequately understood.

A significant knowledge deficit exists in the comprehensive elucidation of these mechanisms and the magnitude of the impact of lead on male reproductive health. Previous research has offered fragmented insights, often constrained by methodological limitations and a narrow focus. Notably, the meta-analysis by Giulioni *et al.* (2023) demonstrated the impact of lead exposure on semen quality; however, the study had some limitations. The number of studies included in the meta-analysis of Giulioni *et al.* (2023) was limited. Also, the quality of evidence and potential biases were not fully explored. Moreover, the quantitative analysis of the interplay between lead-induced oxidative stress and hormonal alterations has not been conducted, leaving a void in comprehension regarding the broader implications for reproductive toxicity.

To bridge this gap, the current study endeavors to undertake a systematic review and meta-analysis of the literature concerning lead exposure and semen quality. By aggregating data from a diverse array of studies, this research aims to provide a more accurate and generalizable estimation of the impact of lead on male reproductive health. Furthermore, it delves into the underlying mechanisms, including lead bioaccumulation, hormone disruption, and oxidative stress.

## MATERIAL AND METHODS

### Search strategy and selection of eligible studies

This is a systematic review and meta-analysis that evaluates the harmful effects of lead exposure on the quality of semen. The scientific literature was systematically searched using Google Scholar, Scopus, and PubMed, between March 18, 2024, and April 30, 2024. These Medical Subject Headings and Boolean operators were used: (male infertility OR male reproductive toxicity OR sperm OR semen) AND (lead OR lead exposure OR Pb). All relevant data were harvested without date and language restrictions.

The Population Exposure Comparison Outcome Study Design (PECOS) model was employed to design the inclusion and exclusion criteria, which were used to screen and identify eligible studies.

Population (P): The study population was men in their reproductive age.

Exposure (E): The studied subjects must have been exposed to lead.

Comparison (C): The lead-exposed population must have been compared with an unexposed population/control. In cases where there were no strict controls, the blood concentrations of lead were used to determine the exposure levels.

Outcome (O): The primary outcomes were ejaculate volume, semen viscosity, sperm count, concentration, morphology (normal and abnormal), vitality, and motility (total and progressive), while the secondary outcomes were blood and semen concentrations of lead, serum FSH, LH, and testosterone levels, and semen malondialdehyde (MDA) concentration.

Study design (S): Observational studies in humans.

Published articles were collected by all authors, while three authors (AVI, ACA, and ATM) independently screened the collected studies for eligibility, and discrepancies were resolved by a fourth author (ARE).

### Appraisal of the quality of the eligible studies and data collation

Two authors (APJ and ATM) independently assessed the eligibility for studies for risk of bias (RoB) and confidence of certainty. A third author (ARE) resolved all discrepancies. The ErasmusAGE quality score for systematic reviews was used to assess the level of evidence in the included papers (Hamed *et al.*, 2023), while the Newcastle-Ottawa Quality Assessment Form for Cohort Studies was used to assess the RoB (Wells *et al.*, 2025), and the degree of confidence in the evidence was established by the Grading of Recommendations Assessment, Development, and Evaluation (GRADE) Working Group recommendations (GRADE, 2014).

### Meta-analysis

Quantitative analyses were done using Review Manager (5.4.1). The standardized mean difference (SMD) and 95% confidence interval (CI) were pooled from the included studies. When there was insignificant variation, a fixed effect (FE) model was employed; otherwise, a random effect (RE) model was used. Significant variation exists when the *p-*value of heterogeneity ≤0.1 or I^2^≥50%, and it is not significant when the *p*-value of heterogeneity >0.1 or I^2^<50%. A statistically significant result was defined as an overall effect value of *p*<0.05. Sensitivity analyses were performed by removing the research with the highest weight and studies with poor evidence quality (<5), high RoB, and low or very low certainty of evidence to analytically assess the robustness of our findings, detect the potential sources of bias, and improve the reliability of the synthesized evidence. Publication bias was examined visually using the funnel plot.

## RESULTS

### Study selection and characteristics of the eligible studies

**Figure 1:**
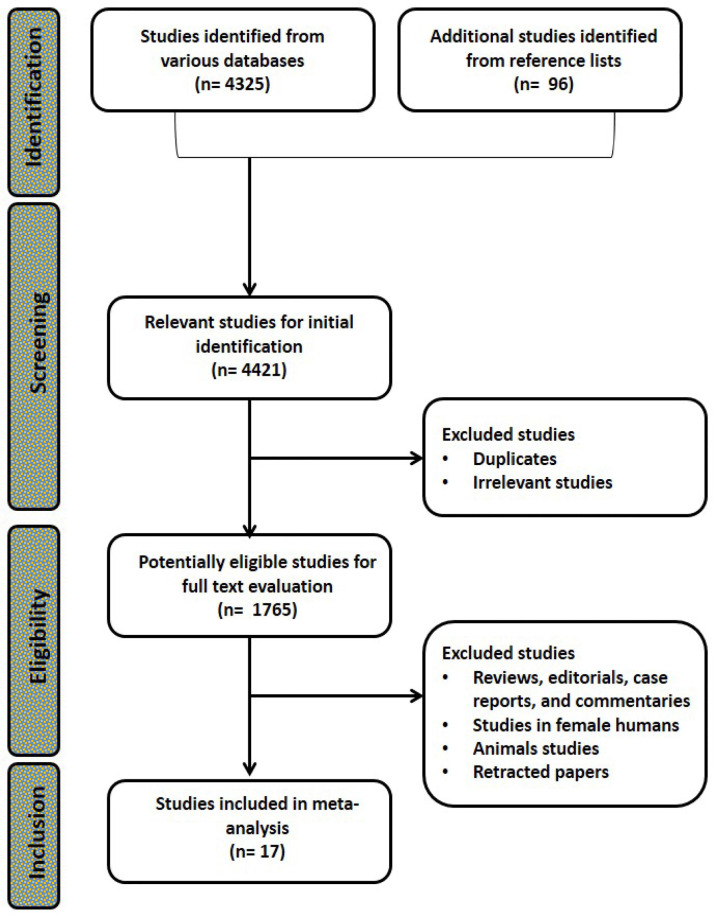
PRISMA flow chart showing the search strategy and identification of studies eligible for inclusion.

The flow chart showing the search strategy and identification of studies eligible for inclusion is presented in Figure 1. The systematic literature search yielded 4325 papers with an additional 96 identified from the reference lists. After excluding duplicates, irrelevant studies, retracted papers, animal studies, studies in females, and reviews, editorials, case reports, and commentaries, 18 studies from 17 papers were deemed eligible for inclusion.

The qualified studies were published between 1996 and 2024. All the studies were cross-sectional. Table 1 also displays additional details about the qualifying studies, including the nation in which the research was carried out, the size of the study population, participant age, exposure length, and the outcome that was measured.

**Table 1:** Characteristics of the eligible studies.

Study	Study design	Country	Studied population	Age (Years)	Exposure	Duration of exposure	Measured outcome
**Alexander** ***et al .,* 1996**	cross sectional	Cominco smelter in Trail, British Columbia.	2469(male workers employed on 1 May, 1993 (n = 2123) and workers laid off in the preceding year (n = 346)	39.7-42.3	Occupational lead exposure, Blood lead concentration.	June and July, 1993	Sperm concentration, motility, total sperm count, testosterone, LH, FSH, Blood and semen lead concentration
**Alexander** ***et al .,* 1998**	cross-sectional	Trail, British Columbia	(n = 2,469)	39.0-40.1	Occupational lead exposure, Blood lead concentration.		Sperm concentration, sperm count, Blood and semen lead concentration
**Bonde** **et al., 2014**	cross-sectional	United Kingdom, Italy, and Belgium	503	35.7-40.0	seminal plasma and spermatozoa	2 YEARS	Sperm concentration, total sperm count, semen volume, blood and semen lead concentration
**Eibensteiner et al., 2005**	cross-sectional study	Arequipa, Peru.	(n = 43)	20-55 years	Blood lead concentration	15 DAYS	Sperm concentration, motility, total sperm count. Morphology, viability, testosterone, concentration
**Farag et al., 2024**	case-control cross-sectional	Egypt	80 adult	30 to 50 years	Blood lead concentration	1 year	Sperm concentration, motility, total sperm count, viability, Blood and semen lead concentration
**Hovatta** **et al., 1998**	cross-sectional retrospective	Helsinki, Finland.	N = 424	20-46	spermatozoa and seminal plasma lead concentration	1 year?	Sperm concentration, motility, morphology
**Hsu et al., 2009**	cross-sectional	Taiwan.	N=80	29.2 + 3.9	Occupational lead exposure, spermatozoa and seminal plasma lead concentration	1.7 year	Sperm concentration, motility, total sperm count, Blood and semen lead concentration
**Kasperczyk et al., 2008**	cross-sectional	Poland	(n= 63)	34-38	Occupational lead exposure, Blood lead concentration	13 years	Sperm concentration, motility, volume total sperm count, Blood and semen lead concentration
**Kuo et al., 1997**	cross-sectional	Taiwan.	N=13		Occupational lead exposure, spermatozoa and seminal plasma lead concentration		viscosity, motility, total sperm count, Blood and semen lead concentration
**Mahmoud** **et al., 2005**	cross-sectional	Belgium	N=159	25-57	Occupational lead exposure, spermatozoa and seminal plasma lead concentration		Sperm concentration, volume, inhibin, FSH, Blood and semen lead concentration
**Moran-Martinez** ***et al*., 2013**	cross-sectional	USA	N=47	20-27 years	Environmental lead exposure, spermatozoa and seminal plasma lead concentration	2 years	Sperm motility, viability, volume, morphology Blood and semen lead concentration
**Naha and Chowdhury, 2006**	cross-sectional	Kolkata, India	N=130	(31-45years	Occupational lead exposure, spermatozoa and seminal plasma lead concentration	group ll: 7 to 1O years (group iii: more than 1O to 15 years	Sperm viability, sperm MDA Blood and semen lead concentration
**Naha and Manna, 2007**	cross-sectional	Bangalore, India	N=100	31-45years	Occupational lead exposure, spermatozoa and seminal plasma lead concentration	group ll: 7 to 1O years (group iii: more than 1O to 15 years	Sperm concentration, motility, viability , volume, morphology testosterone, LH, FSH, Blood and semen lead concentration
**Naha *et al*., 2005**	cross-sectional	Kolkata, India	N=120	31-45years	Occupational lead exposure, spermatozoa and seminal plasma lead concentration	7-15 years exposure	Sperm concentration, motility, Viability, volume, viscosity, total sperm count, , Blood and semen lead concentration
**Saaramen *et al*., 1987**	cross-sectional	Kuopio, finland	N=160	22-47years	Environmental lead exposure, spermatozoa and seminal plasma lead concentration		Sperm morphology, motility, density, Blood and semen lead concentration
**Telisman *et al*.,2000**	cross-sectional	Zagreb, Croatia	N=149	20-43years	Environmental lead exposure, spematozoa and seminal plasma lead concentration	15years	Sperm concentration, motility, density, volume,morphology total sperm count, testosterone, LH, FSH, Blood and semen lead concentration
**Wijesekara *et al.*, 2020.**	cross-sectional	Colombo	(n = 300)	34.9 (5.30) 34.8 (5.37)	spematozoa and seminal plasma lead concentration		Sperm concentration, motility, total sperm count, progressive motility Blood and semen lead concentration

### Study quality

The quality of evidence scores for each domain and the global score are shown in Table 2. All the eligible studies, except Morán-Martínez *et al.* (2013), showed a good quality of evidence. Two studies (Eibensteiner *et al.*, 2005; Farag *et al.*, 2024) had a marginal quality of evidence. The study design domain had a low score in all the papers since all the eligible studies were cross-sectional.

**Table 2:** Assessment of the quality of evidence.

Study	Study design	Study size	Methods of measuring exposure	Method of measuring outcome	Analysis with adjustment	Total
**Alexander** ***et al*., 1996**	0	2	2	2	1	7/10
**Alexander** ***et al*., 1998**	0	2	2	2	2	8/10
**Bonde** ***et al*., 2014**	0	2	2	2	2	8/10
**Eibensteiner** ***et al*., 2005**	0	0	2	2	1	5/10
**Farag** ***et al*., 2024**	0	1	2	2	0	5/10
**Hovatta** ***et al*., 1998**	0	2	2	2	2	8/10
**Hsu** ***et al*., 2009**	0	1	2	2	2	7/10
**Kasperczyk** ***et al*., 2008**	0	1	2	2	1	6/10
**Kuo** ***et al*., 1997**	0	0	2	2	2	6/10
**Mahmoud** ***et al*., 2005**	0	2	2	2	2	8/10
**Moran-Martinez** ***et al*., 2013**	0	0	2	2	0	4/10
**Naha and Chowdhury, 2006**	0	1	2	2	2	7/10
**Naha and Manna, 2007**	0	1	2	2	2	7/10
**Naha** ***et al*., 2005**	0	1	2	2	2	7/10
**Saaramen** ***et al*., 1987**	0	2	2	2	0	6/10
**Telisman** ***et al*.,2000**	0	1	2	2	2	7/10
**Wijesekara** ***et al*., 2020.**	0	2	2	2	0	6/10

Studies with values of at least 5 are of good quality of evidence.

Table 3 shows the RoB of the included studies. Overall, all studies had a moderate to low RoB. The selection of the exposed cohort and assessment of exposure had a low RoB (1 star) in all the included studies. More so, four studies (Saaranen *et al.*, 1987; Alexander *et al.*, 1996; Kuo *et al.*, 1997; Eibensteiner *et al.*, 2005) had a low certainty of evidence, while ten studies (Alexander *et al.*, 1998; Hovatta *et al.*, 1998; Telisman *et al.*, 2000; Bonde *et al.*, 2002; Mahmoud *et al.*, 2005; Kasperczyk *et al.*, 2008; Hsu *et al.*, 2009; Morán-Martínez *et al.*, 2013; Wijesekara *et al.*, 2020; Farag *et al.*, 2024) had a moderate certainty of evidence, and three studies (Naha *et al.*, 2005; Naha & Chowdhury, 2006; Naha & Manna, 2007) had a high certainty of evidence (Table 4).

**Table 3:** Assessment of the risk of bias.

Study	Selection of exposed cohort	Selection of non-exposed cohort	Assessment of exposure	Demonstration that outcome was not present at the start of the study	Comparability (basics)	Comparability (others)	Assessment of outcome	Length of follow up	Adequacy of follow up	Total
**Alexander** ***et al*., 1996**	1	0	1	0	1	1	1	0	0	5/9
**Alexander** ***et al*., 1998**	1	0	1	0	1	1	1	0	0	5/9
**Bonde** ***et al*., 2014**	1	0	1	0	1	1	1	0	0	5/9
**Eibensteiner *et al*., 2005**	1	0	1	0	1	1	1	0	0	5/9
**Farag** ***et al*., 2024**	1	1	1	0	1	0	1	0	0	5/9
**Hovatta *et al*., 1998**	1	0	1	0	1	1	1	0	0	5/9
**Hsu** ***et al*., 2009**	1	0	1	0	1	1	1	0	0	5/9
**Kasperczyk** ***et al*., 2008**	1	1	1	0	1	1	1	0	0	6/9
**Kuo** ***et al*., 1997**	1	1	1	0	1	1	1	0	0	6/9
**Mahmoud** ***et al*., 2005**	1	1	1	0	1	1	1	0	0	6/9
**Moran-Martinez** ***et al*., 2013**	1	1	1	0	1	0	1	0	0	5/9
**Naha and Chowdhury,** **2006**	1	1	1	0	1	1	1	0	0	6/9
**Naha and Manna, 2007**	1	1	1	0	1	1	1	0	0	6/9
**Naha** ***et al*., 2005**	1	1	1	0	1	1	1	0	0	6/9
**Saaramen** ***et al*., 1987**	1	0	1	0	1	0	1	0	0	4/9
**Telisman** ***et al*.,2000**	1	1	1	0	1	1	1	0	0	6/9
**Wijesekara** ***et al*., 2020.**	1	1	1	0	1	0	1	0	0	5/9

Number indicates the number of stars

6-9: Low risk of bias, 4 and 5: moderate risk of bias, ≤ 3: high risk of bias

**Table 4:** Assessment of the confidence in the body of evidence (certainty of evidence).

Study	Initial Confidence	Decreasing	Increasing	Final Confidence
**Alexander et al ., 1996**	Low	Yes, 2	Yes, 2	low
**Alexander *et al*., 1998**	Low	Yes, 1	Yes, 2	Moderate
**Bonde *et al*., 2014**	Low	Yes, 1	Yes, 3	moderate
**Eibensteiner *et al*., 2005**	Low	Yes, 1	Yes, 1	low
**Farag *et al*., 2024**	Low	Yes, 1	Yes, 2	Moderate
**Hovatta *et al*., 1998**	Low	Yes, 1	Yes, 2	Moderate
**Hsu *et al*., 2009**	Low	Yes, 1	Yes, 2	Moderate
**Kasperczyk *et al*., 2008**	Moderate	Yes, 1	Yes, 2	Moderate
**Kuo *et al*., 1997**	Moderate	Yes, 2	Yes, 1	Low
**Mahmoud *et al*., 2005**	Moderate	Yes, 1	Yes, 1	Moderate
**Moran-Martinez *et al*., 2013**	Moderate	Yes, 1	Yes, 1	Moderate
**Naha and Chowdhury, 2006**	Moderate	N0	Yes, 1	High
**Naha and Manna, 2007**	Moderate	N0	Yes, 1	High
**Naha *et al*., 2005**	Moderate	N0	Yes , 1	High
**Saaramen *et al*., 1987**	Low	NO	no	low
**Telisman *et al*., 2000**	Moderate	Yes , 1	Yes , 2	Moderate
**Wijesekara *et al*., 2020**	Moderate	Yes , 1	NO	Moderate

### Quantitative analysis

#### Lead concentrations in the blood and semen

**Figure 2:**
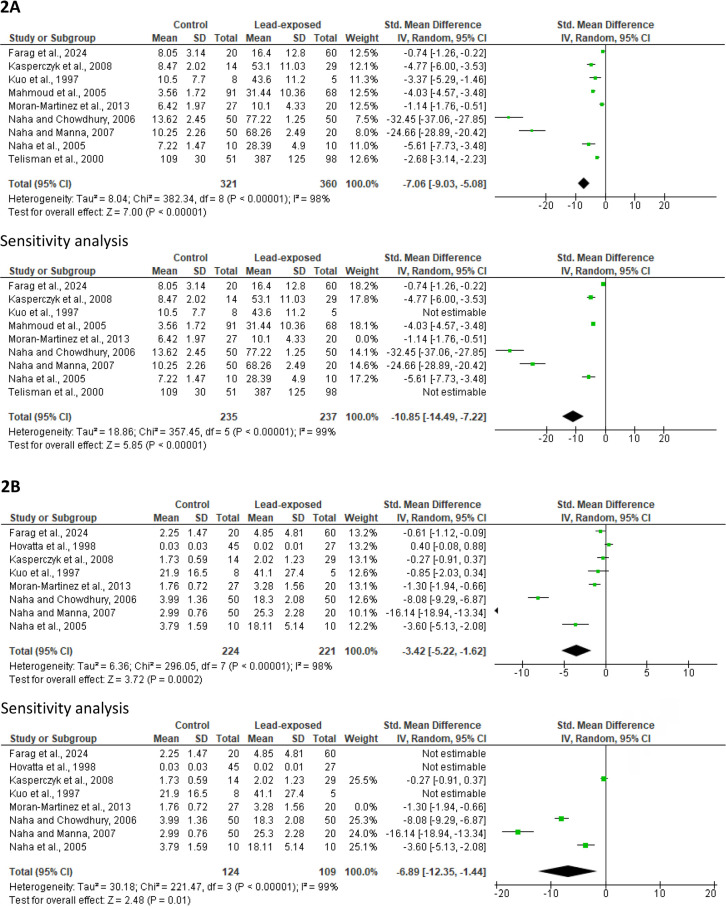
Effect of lead exposure on blood lead concentration (A) and semen lead concentration (B). Values are shown as standardized mean difference and 95% confidence interval.

**Supplementary Figure 1:**
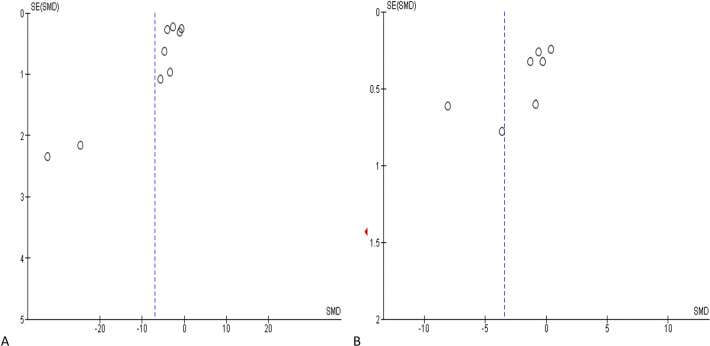
Publication bias of the studies on the effect of lead exposure on blood lead concentration (A) and semen lead concentration (B).

Nine studies were included in the evaluation of blood lead concentration, which included 360 cases of lead-exposed men and 321 controls. Blood lead level was significantly increased in lead-exposed men more than in the control (SMD -7.06 [95% CI: -9.03, -5.08] *p*<0.00001). The study heterogeneity was significant (I^2^ = 98%; *X*^2^ *p*<0.00001). After sensitivity analysis, the significant difference in blood lead concentration between the control and lead-exposed men persisted (SMD -10.85 [95% CI: -14.49, -7.22] *p*<0.00001), with a considerable study heterogeneity (I^2^ = 99%; *X*^2^ *p*<0.00001) (Fig. 2A). There was no evidence of publication bias. (Supplementary Fig. 1A).

Quantitative analysis of the eight studies included in the evaluation of semen lead concentration revealed that exposure to lead led to a significant rise in semen lead levels when compared with the controls (SMD -3.42 [95% CI: -5.22, -1.62] *p*=0.0002). There was a significant inter-study diversity (I^2^ = 98%; *X*^2^ *p*<0.00001). After sensitivity analysis, semen lead concentration was yet significantly higher in lead-exposed men than in the controls (SMD -6.89 [95% CI: -12.35, -1.44] *p=*0.01), and there was also a considerable study heterogeneity (I^2^ = 99%; *X*^2^ *p<*0.00001) (Fig. 2B). There was no evidence of publication bias (Supplementary Fig. 1B).

#### Semen viscosity

**Supplementary Figure 2:**
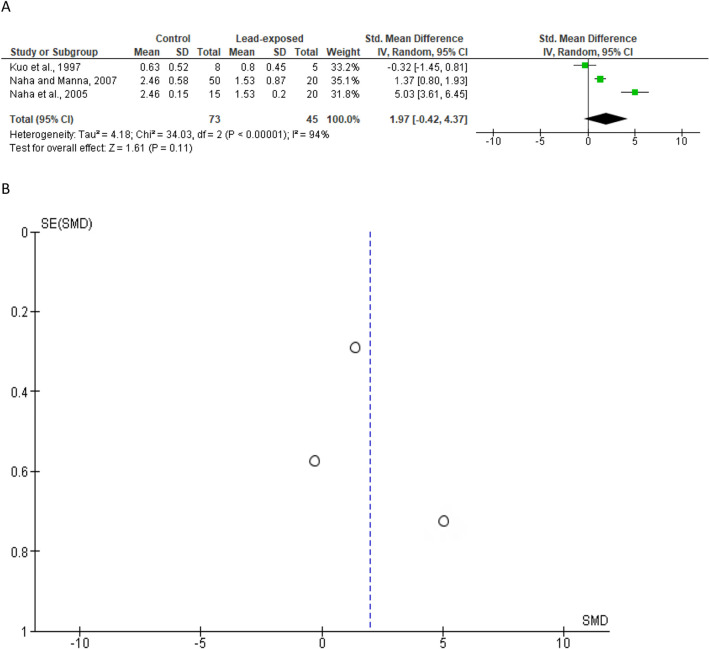
Effect of lead exposure on semen viscosity (A) and the publication bias of the studies on semen viscosity (B).

The meta-analysis assessing the effect of exposure to lead on semen viscosity only had three studies: 73 control and 45 lead-exposed men. No significant difference was found in the semen viscosity of men exposed to lead when compared with the control group (SMD 1.97 [95% CI: -0.42, 4.37], *p=*0.11). There was a significant inter-study diversity (I^2^ = 94%; *X*^2^ *p*<0.00001) (Supplementary Fig. 2A). There was a considerable publication bias (Supplementary Fig. 2B).

#### Ejaculate volume

**Figure 3:**
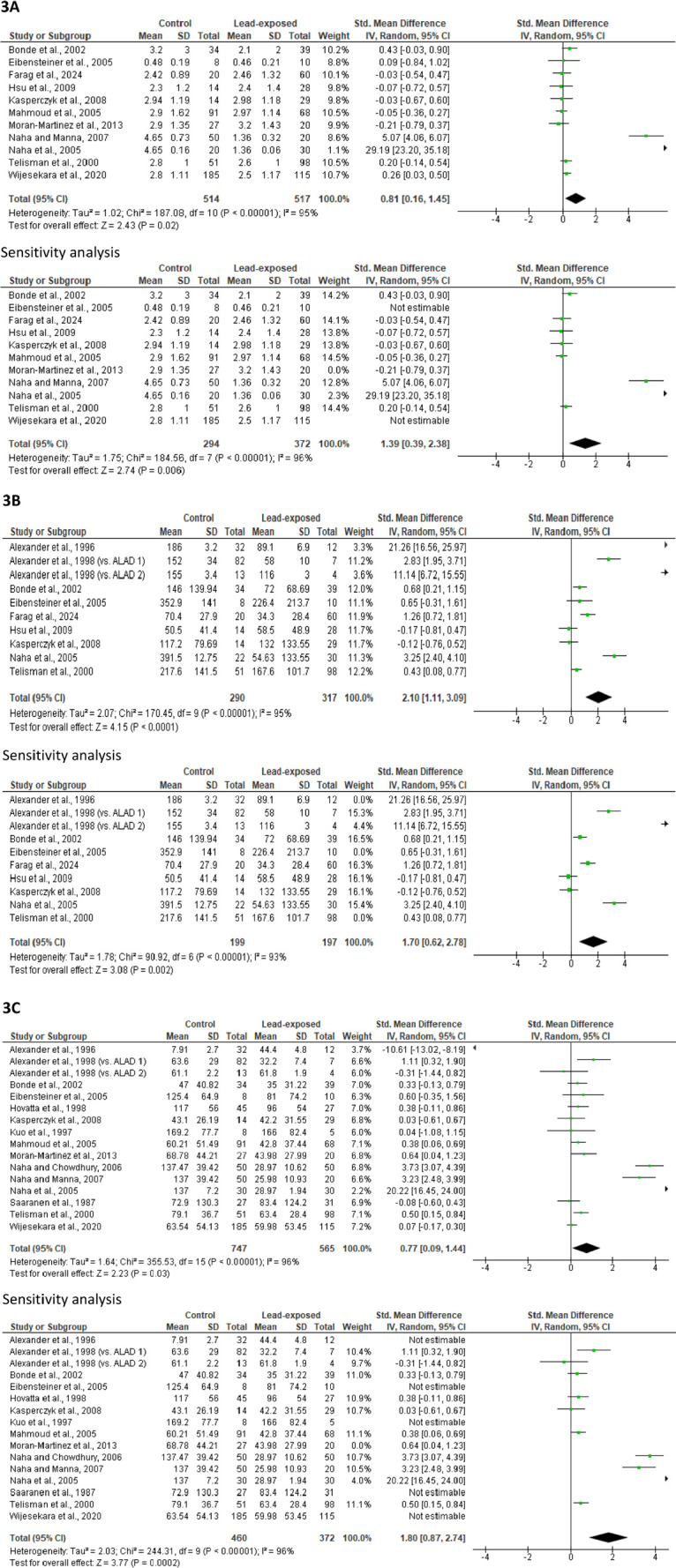
Effect of lead exposure on ejaculate volume (A), sperm count (B), and sperm concentration (C). Values are shown as standardized mean difference and 95% confidence interval.

**Supplementary Figure 3:**
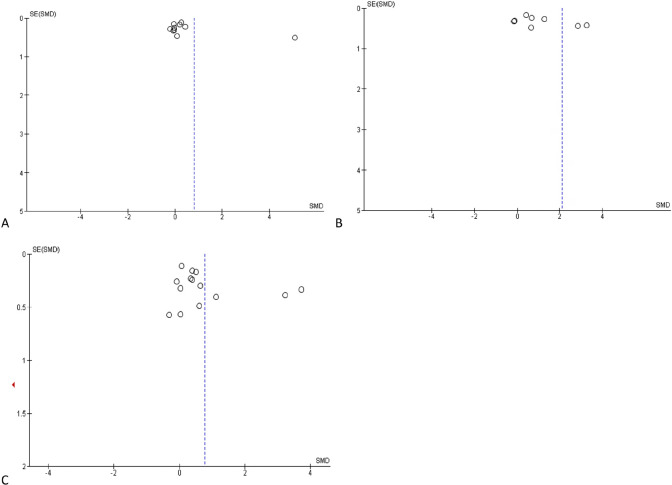
Publication bias of the studies on the effect of lead exposure on ejaculate volume (A), sperm count (B), and sperm concentration (C).

Quantitative analysis of eleven studies (514 control *versus* 517 lead-exposed) showed that the control subjects had a significantly higher ejaculate volume than the lead-exposed men (SMD 0.81 [95% CI: 0.16, 1.45] *p*=0.02), with a considerable study heterogeneity (I^2^ = 95%; *X*^2^ *p*<0.00001). After sensitivity analysis, ejaculate volume was still significantly higher in the control than in the lead-exposed men (SMD 1.39 [95% CI: 0.39, 2.38] *p*=0.006). This showed a significant study heterogeneity (I^2^ = 96%; *X*^2^ *p*<0.00001) (Fig. 3A). There was a noticeable publication bias (Supplementary Fig. 3A).

#### Sperm count

Meta-analysis of ten studies from nine articles (290 control *versus* 317 lead-exposed) demonstrated a significantly lower sperm count in lead-exposed men compared with the control (SMD 2.10 [95% CI: 1.11, 3.09], *p*<0.0001). There was a considerable inter-study diversity (I^2^ = 95%; *X*^2^ *p*<0.00001). After sensitivity analysis, the significant reduction in sperm count following lead exposure persisted (SMD 1.70 [95% CI: 0.62, 2.78], *p*=0.002). This also revealed a significant study heterogeneity (I^2^ = 93%; *X*^2^ *p*<0.00001) (Fig. 3B). Also, a publication bias was observed (Supplementary Fig. 3B).

#### Sperm concentration

Quantitative analysis of sixteen studies (747 control *versus* 565 lead-exposed) showed that lead exposure led to a significantly reduced sperm concentration when compared with the control (SMD 0.77 [95% CI: 0.09, 1.44], *p*=0.03). There was a substantial heterogeneity (I^2^ = 96%; *X*^2^ *p*<0.00001). More so, a sensitivity analysis of these studies revealed a reduced sperm concentration in lead-exposed men when compared with the control (SMD 1.80 [95% CI: 0.87, 2.74] *p*=0.0002), with a considerable study heterogeneity (I^2^ = 96%; *X*^2^ *p*<0.00001) (Fig. 3C). There was a substantial publication bias (Supplementary Fig. 3C).

#### Sperm vitality

**Figure 4:**
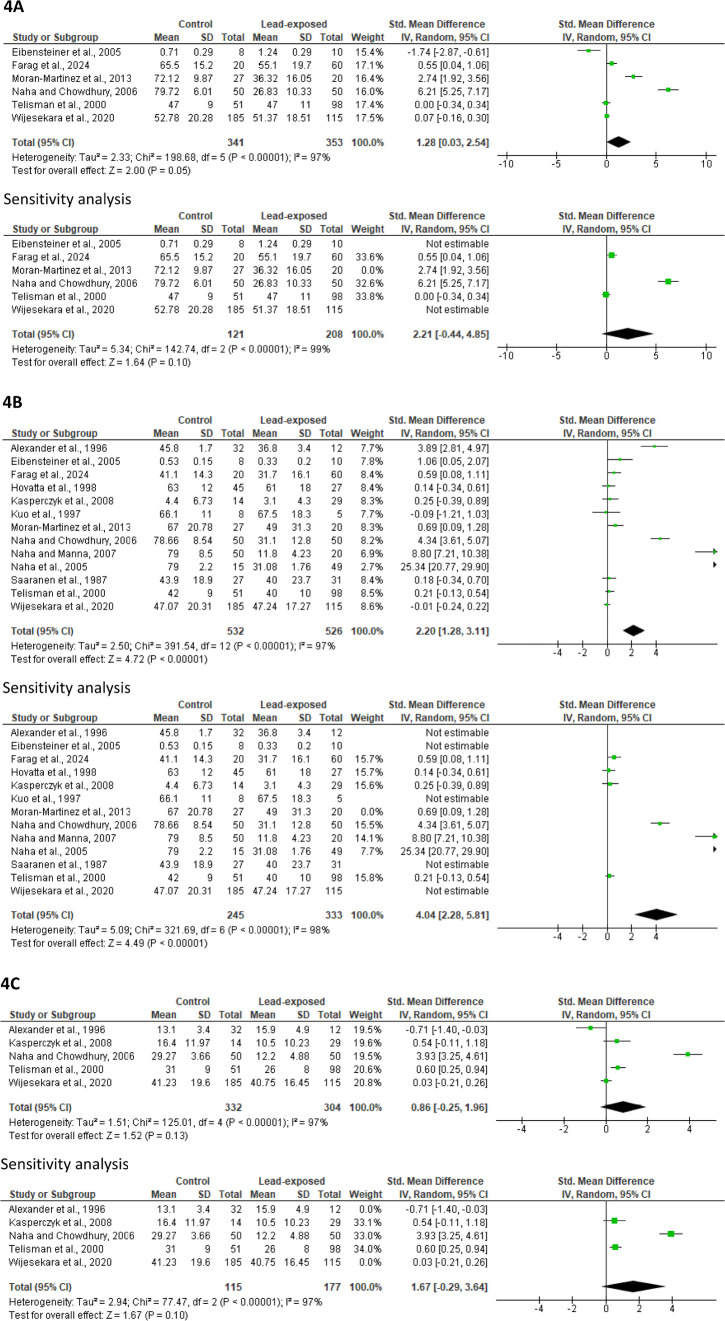
Effect of lead exposure on sperm vitality (A), total motility (B), and progressive motility. Values are shown as standardized mean difference and 95% confidence interval.

**Supplementary Figure 4:**
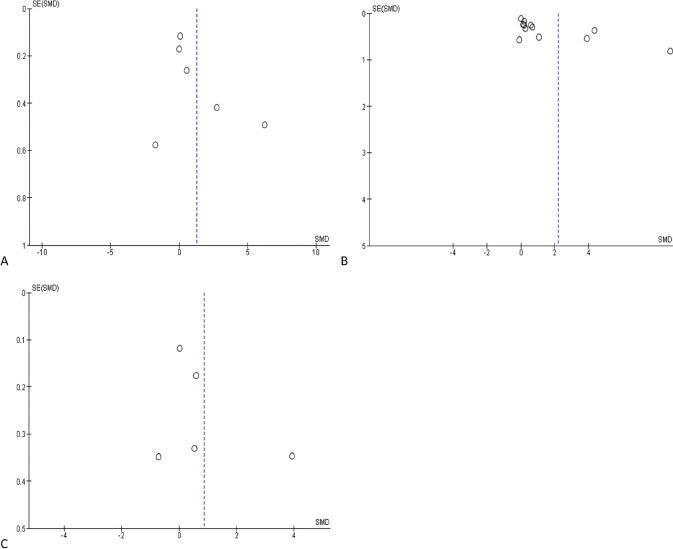
Publication bias of the studies on the effect of lead exposure on sperm vitality (A), total motility (B), and progressive motility (C).

Meta-analysis of six studies (341 control *versus* 353 lead-exposed) showed a marginal but not significant decline in sperm vitality in lead-exposed men when compared with the control (SMD 1.28 [95% CI: 0.03, 2.54] *p*=0.05) with a significant study heterogeneity (I^2^ = 97%; *X*^2^ *p*<0.00001), which persisted following a sensitivity analysis (SMD 2.21 [95% CI: -0.44, 4.85] *p*=*p*0.10) with a significant study heterogeneity (I^2^ = 99%; *X*^2^ *p*<0.00001) (Fig. 4A). A publication bias was observed (Supplementary Fig. 4A).

#### Sperm total and progressive motility

Meta-analysis of thirteen studies (532 control *versus* 526 lead-exposed) showed a significantly lower sperm total motility in lead-exposed men compared with the control (SMD 2.20 [95% CI: 1.28, 3.11] *p*<0.00001) and a significant study heterogeneity (I^2^ = 97%; *X*^2^ *p*<0.00001). After a sensitivity analysis, sperm total motility was still significantly reduced in lead-exposed men when compared with the control (SMD 4.04 [95% CI: 2.28, 5.81] *p*<0.00001) and there was a significant inter-study diversity (I^2^ = 98%; *X*^2^ *p<*0.00001) (Fig. 4B). There was a noticeable publication bias (Supplementary Fig. 4B).

Quantitative analysis on sperm progressive motility included five studies which involved 332 control and 304 lead-exposed men. There was no significant alteration in the sperm progressive motility between the control and the lead-exposed groups (SMD 0.86 [95% CI: -0.25, 1.96] *p=p*0.13) and there was a considerable study heterogeneity (I^2^ = 97%; *X*^2^ *p*<0.00001) even after a sensitivity analysis (SMD 1.67 [95% CI: -0.29, 3.64] *p*=0.10; I^2^ = 97%; *X*^2^ *p*<0.00001) (Fig. 4C). Publication bias was observed (Supplementary Fig. 4C).

#### Sperm morphology

**Figure 5:**
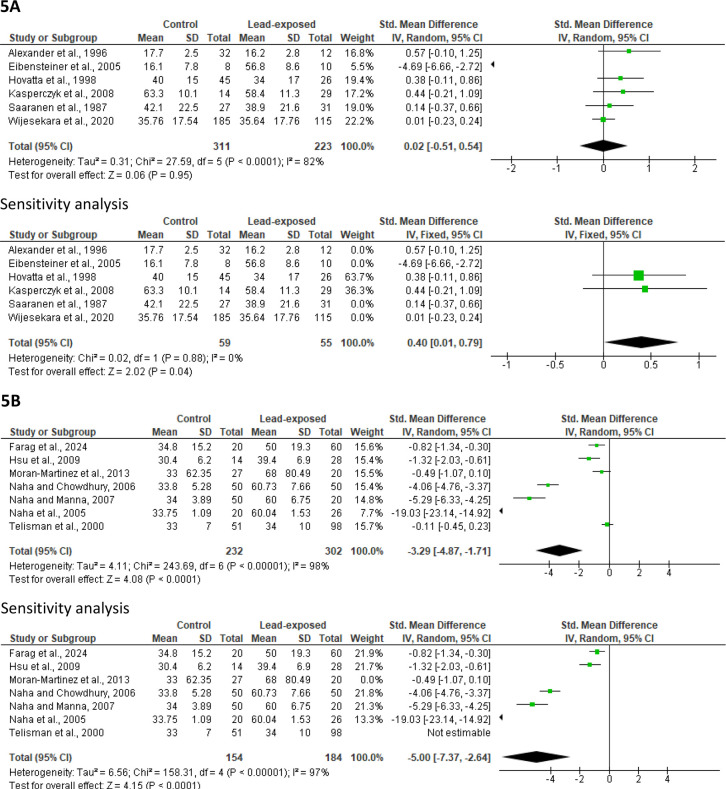
Effect of lead exposure on sperm normal morphology (A) and abnormal morphology (B). Values are shown as standardized mean difference and 95% confidence interval.

**Supplementary Figure 5:**
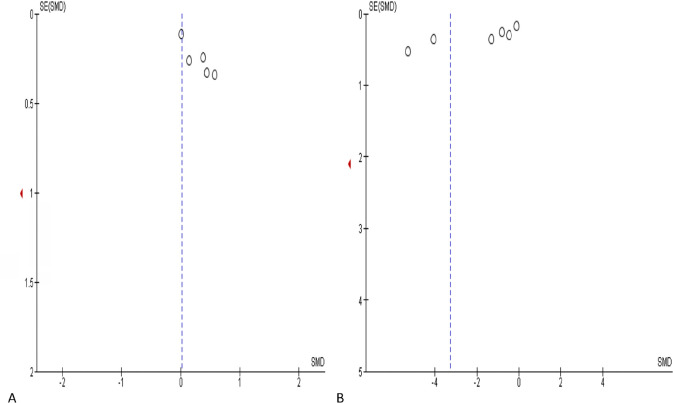
Publication bias of the studies on the effect of lead exposure on sperm normal morphology (A) and abnormal morphology (B).

An analysis of six trials comparing 223 men exposed to lead and 311 control men was conducted to determine the effect of lead exposure on normal sperm morphology. Lead exposure did not alter sperm normal morphology (SMD 0.02 [95% CI: -0.51, 0.54] *p*=0.95), and there was substantial study heterogeneity (I^2^ = 82%; *X*^2^ *p*<0.0001). However, after a sensitivity analysis, lead exposure was observed to significantly reduce sperm normal morphology when compared with the control (SMD 0.40 [95% CI: 0.01, 0.79] *p*=0.04) and there was no considerable study heterogeneity (I^2^ = 0%; *X*^2^ *p*=0.88) (Fig. 5A). A publication bias was noticed (Supplementary Fig. 5A).

Additionally, a meta-analysis of seven studies (232 control *versus* 302 lead-exposed) demonstrated a significant increase in sperm abnormal morphology in lead-exposed men when compared with the control (SMD -3.29 [95% CI: -4.87, -1.71] *p*<0.0001) with a significant inter-study diversity (I^2^ = 98%; *X*^2^ *p*<0.00001). After a sensitivity analysis, the observed significant rise in sperm abnormal morphology in men exposed to lead in comparison with the control persisted (SMD -5.00 [95% CI: -7.37, -2.64] *p*<0.0001) with a significant inter-study diversity (I^2^ = 97%; *X*^2^ *p*<0.00001) (Fig. 5B). Publication bias was observed (Supplementary Fig. 5B).

#### Hormone profile

**Figure 6:**
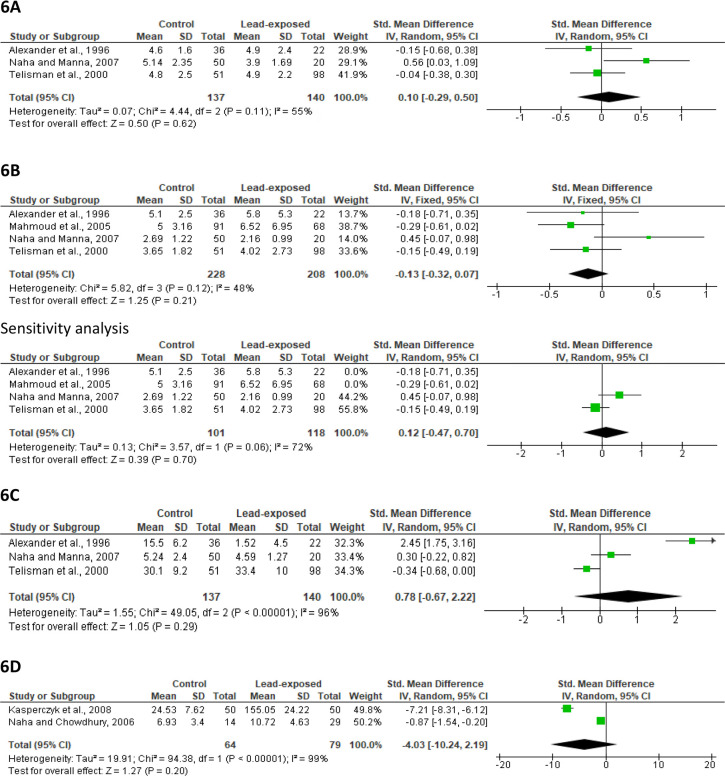
Effect of lead exposure on serum LH (A), FSH (B), testosterone (C), and semen MDA concentration (D). Values are shown as standardized mean difference and 95% confidence interval.

**Supplementary Figure 6:**
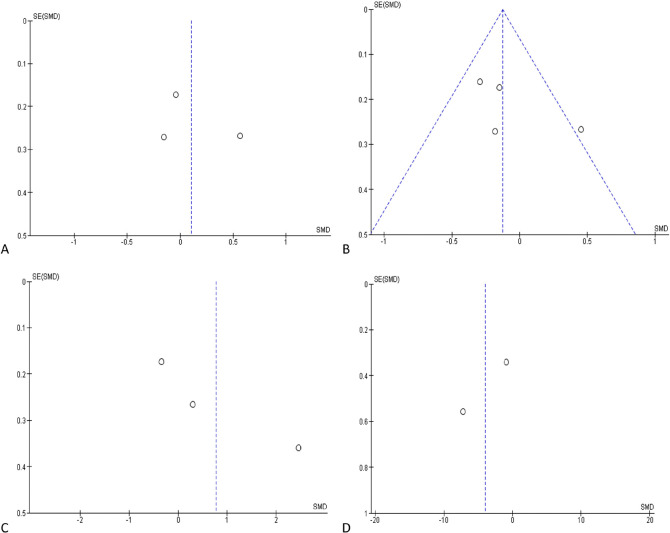
Publication bias of the studies on the effect of lead exposure on serum LH (A), FSH (B), testosterone (C), and semen MDA level (D).

Meta-analysis of the three studies included (137 control *versus* 140 lead-exposed) revealed that lead exposure did not significantly alter serum LH concentration (SMD 0.10 [95% CI: -0.29, 0.50] *p*=0.62). There was significant study heterogeneity (I^2^ = 55%; *X*^2^ *p*=0.11) (Fig. 6A). Publication bias was observed (Supplementary Fig. 6A).

In addition, a meta-analysis of four studies (228 control *versus* 208 lead-exposed) demonstrated that lead exposure did not significantly alter serum FSH levels (SMD -0.13 [95% CI: -0.32, 0.07] *p=*0.21) and inter-study diversity was not observed (I^2^ = 48%; *X*^2^ *p*=0.12). After a sensitivity analysis, there was yet no significant alteration in serum FSH levels between the lead-exposed and control (SMD 0.12 [95% CI: -0.47, 0.70] *p*=0.70), and significant inter-study diversity was observed (I^2^ = 72%; *X*^2^ *p*=0.06) (Fig. 6B). Publication bias was also observed (Supplementary Fig. 6B).

Quantitative analysis of three studies (137 control *versus* 140 lead-exposed) showed no significant difference in serum testosterone concentration in men exposed to lead when compared with the control (SMD 0.78 [95% CI: -0.67, 2.22] *p*=0.29). There was a significant study heterogeneity (I^2^ = 96%; *X*^2^ *p*=0.29) (Fig. 6C), and publication bias was also observed (Supplementary Fig. 6C).

#### Semen MDA

Meta-analysis of two studies (64 control *versus* 79 lead-exposed) revealed that although lead exposure caused a rise in semen MDA level when compared with the control, this was not significant statistically (SMD -4.03 [95% CI: -10.24, 2.19] *p*=0.20) and a significant study heterogeneity was observed (I^2^ = 99%; *X*^2^ *p<*0.00001) (Fig. 6D). There was no publication bias (Supplementary Fig. 6D).

## DISCUSSION

This study not only demonstrated the toxic effect of exposure to lead on semen quality, but it also revealed the involvement of lead bioaccumulation, male reproductive hormones (LH, FSH, and testosterone), and oxidative stress in lead-induced alteration of semen quality. We observed that exposure to lead significantly reduced total motility, normal morphology, ejaculate volume, sperm concentration, and count, but increased abnormal morphology, which was accompanied by a rise in serum and semen lead concentrations.

Findings from this study revealed that lead-induced decline in semen quality may involve several mechanistic pathways. Lead exposure promotes lead accumulation in the serum and semen and may directly induce testicular injury. Lead may disrupt the blood-testis barrier and blood-epididymis barrier, thus promoting its accumulation in these tissues where it impairs spermatogenesis and induces direct injury to germ cells and spermatozoa, leading to reduced total motility, normal morphology, ejaculate volume, sperm concentration, and count. This agrees with the outcomes in experimental studies (Batra *et al.*, 2001; Gorbel *et al.*, 2002) that revealed that lead accumulation disrupts spermatogenesis and damages germ cells.

Although the decline observed in the serum LH and testosterone levels following lead exposure was not statistically significant, it shows that lead exposure may potentially impair testosterone biosynthesis by suppressing the hypothalamic-pituitary axis and/or through the induction of a direct injury to the testis and testicular cells such as the Leydig cells, thus reducing the circulating levels of testosterone. Since optimal levels of testosterone are essential in spermatogenesis (Akhigbe *et al.*, 2024a; Saka *et al.*, 2024), lead-induced suppression of testosterone levels may impair spermatogenesis, which explains the decline in ejaculate volume, sperm count, and concentration.

Furthermore, the marginal rise in MDA levels in the seminal fluid in lead-exposed men is a pointer that lead exposure may trigger oxidative stress, which is initiated by ROS generation and the inactivation of glutathione’s sulfhydryl group that has been connected to lead-induced male reproductive harm (Ommati *et al.*, 2023). Oxidative stress is the result, as this disrupts the oxidant/antioxidant equilibrium, suppressing the antioxidant system and causing a redox imbalance (Famurewa *et al.*, 2023). Lead can cause oxidative stress even though it is not a Fenton reaction heavy metal because it indirectly elevates the level of free ferrous (Fe^2+^), a Fenton reaction metal that generates hydroxyl radicals and other free radicals (Farinelli *et al.*, 2020). Lead may also cause calcium, copper, and zinc ions to lose their normal functions as membrane signal transducers or cofactors for antioxidant enzymes, hence increasing the creation of ROS from the beginning (Vukelić *et al.*, 2023). Due to the high polyunsaturated fatty acid content, which makes them more susceptible to oxidative damage, the testis and sperm cells are particularly very responsive to lead and its oxidative stress mediators (Vukelić *et al.*, 2023; Akhigbe *et al.*, 2024a). This may, at least in part, explain the observed lead-induced toxicity on semen quality.

The testes and epididymis contain pattern recognition receptors (PRRs) that activate inflammatory pathways in response to excessive ROS. These PRRs may stimulate the development of inflammatory mediators and, in turn, increase ROS production (Dutta *et al.*, 2021). Several experimental studies (Besong *et al.*, 2023a; 2023b; 2024) revealed that lead exposure upregulated pro-inflammatory cytokines like TNF-α and IL-6, suggesting that lead induces inflammatory responses. The oxidative milieu triggered by lead bioaccumulation may also activate NF-κB signaling, promoting the transcription of mRNA transcripts that encode TNF-α and IL-6 (Akhigbe *et al.*, 2024a, 2024b; 2024c). It has been documented that lead-induced reproductive damage is accompanied by oxidative stress, inflammation, and apoptosis. The dual effect of reactive oxygen species (ROS) and/or oxidative stress could accelerate the death of testicular and germ cells (Besong *et al.*, 2023b) by causing cytochrome C to leak into the cytosol (Kolawole *et al.*, 2022).

Available evidence from the literature also reveals that lead toxicity may be mediated by endoplasmic stress (ER)-driven apoptosis and autophagy via the modulation of ER stress-related genes [Glucose regulator protein 78 and 94 (GRP 78, GRP 94), activating transcription factor 4 and 6 (ATF4, and ATF6)] (Rana, 2020). In response to exposure to heavy metals, GRP 78 and immunoglobin protein (BiP), a master ER chaperone, stimulate macrophage activation and control calcium flux and protein folding. ER stress induces apoptosis of spermatocytes by activating the IRE1-JNK/PERK pathway (Kim *et al.*, 2013), thus impairs fertility.

**Figure 7:**
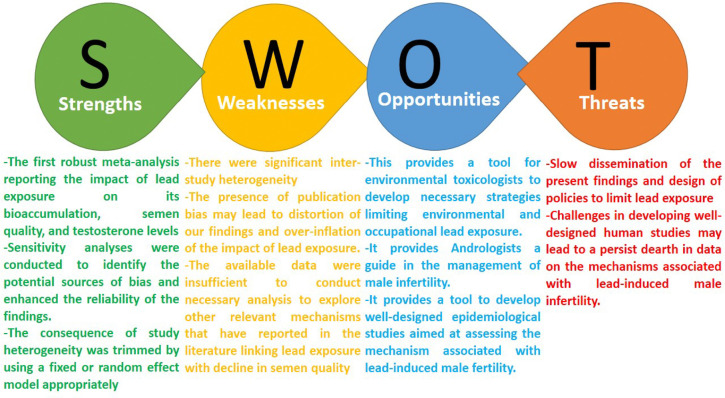
The strengths, weaknesses, opportunities, and threats (SWOT) analysis of the present meta-analysis.

Despite the robustness of this study and the credibility of the evidence synthesized, it has its limitations. First, the presence of significant study heterogeneity might influence our findings. It is likely that the heterogeneity may be due to the study design (all were cross-sectional and observational), varying methods, exposure level and duration, and outcome measurements. Also, the presence of publication bias may lead to distortion of our findings and over-inflation of the impact of lead exposure. Lastly, available data were insufficient to conduct the necessary analysis to explore other relevant mechanisms that have been reported in the literature linking lead exposure with a decline in semen quality. Nonetheless, the strength of this study hinges on several factors. Firstly, it appears that this is the first thorough meta-analysis proving the harmful effects of exposure to lead on semen quality and bioaccumulation, which adds to the mounting evidence of emerging risk factors of male infertility. In addition, the negative impact of the observed study heterogeneity was trimmed by the use of a RE model when a considerable study heterogeneity existed and FE when no study heterogeneity existed. Also, the influence of publication bias was reduced by the performance of a sensitivity analysis, which identified the potential sources of bias and enhanced the reliability of the findings. More so, this study assessed the quality of evidence, RoB, and certainty of confidence in the involved studies. This buttresses the arguments presented in this study. Finally, although not all mechanisms were explored, this study quantitatively assessed the role of lead bioaccumulation, male reproductive hormone disruption, and oxidative stress in lead-induced decline in semen quality (Fig. 7).

Summarily, this study demonstrates that lead exposure significantly reduces ejaculate volume, sperm concentration, total motility, count, and normal morphology but increases abnormal morphology, which is mediated by increased lead bioaccumulation. Thus, necessary strategies to limit environmental and occupational lead exposure should be promoted. In addition, experimental and clinical studies investigating possible interventions that may limit lead bioaccumulation are recommended.
